# Development of phenotypic and genotypic methods for the identification of NmcA β-lactamases in carbapenem-resistant *Enterobacter* spp. and *Klebsiella aerogenes*

**DOI:** 10.1128/spectrum.02675-24

**Published:** 2025-07-31

**Authors:** Ryuichi Nakano, Akiyo Nakano, Yuki Suzuki, Kazuya Narita, Takahiro Sekine, Hisakazu Yano

**Affiliations:** 1 Department of Microbiology and Infectious Diseases, Nara Medical University 12967, Kashihara, Nara, Japan; 2 Division of Central Clinical Laboratory, Iwate Medical University Hospital, Yahaba-cho, Iwate, Japan; University of Kentucky, Lexington, Kentucky, USA

**Keywords:** NmcA, carbapenemase, disc diffusion assay, loop-mediated isothermal amplification (LAMP) assay, *Enterobacter *spp.

## Abstract

Enterobacterales resistant to carbapenems owing to their acquisition of carbapenemase genes have been reported worldwide. *Enterobacter cloacae* complex produces chromosomally encoded inducible Ambler class C β-lactamase but is usually susceptible to carbapenems. However, some studies have reported the incorporation of the NmcA carbapenemase gene, the only inducible carbapenemase classified as Ambler class A, into the chromosomes of members of this complex. Although only a few cases showing NmcA production have been reported, detection methods are urgently needed. Therefore, we developed a double-disk synergy test and loop-mediated isothermal amplification method to detect NmcA producers. The double-disk synergy test was performed by placing a disk of carbapenem (meropenem) 20 mm (center to center) from the disk of a third-generation cephalosporin (cefpodoxime). The loop-mediated isothermal amplification primers were designed to specifically recognize *bla*_NmcA_. NmcA-producing *Enterobacter ludwigii* isolate NR1491 was used as a positive control. The double-disk synergy test results revealed a D-shaped inhibition zone around the cefpodoxime disk, whereas the inhibition zone adjacent to the meropenem disk was blunted. No other D-shaped inhibition zones were observed for other types of carbapenemase producers. The loop-mediated isothermal amplification assay successfully detected NmcA and IMI producers with high homology to NmcA. The detection limit for this assay was 10^−1^ CFU per tube, which is 100-fold more sensitive than the PCR assay for *bla*_NmcA_ detection. The accuracy, sensitivity, and specificity of these assays are high, and these tests are expected to be used for the detection of NmcA producers in clinical laboratories.

**IMPORTANCE:**

NmcA, which is associated with carbapenem resistance, has occasionally been detected in the *Enterobacter cloacae* complex. NmcA is the only inducible carbapenemase whose expression is regulated by transcriptional regulator genes. We developed phenotypic and genotypic methods for the identification of NmcA β-lactamases and evaluated their usefulness in clinical laboratories. The multiplex double-disk synergy test method was developed as a phenotypic method to identify NmcA producers by confirming the phenomenon (induction mechanism) exclusive to NmcA producers. We also developed a loop-mediated isothermal amplification assay as a genotyping method to detect NmcA producers by specifically recognizing *bla*_NmcA_. These methods demonstrated high accuracy, sensitivity, and specificity and are expected to be applied for the detection of NmcA producers in clinical settings.

## INTRODUCTION

*Enterobacter* spp. are clinically significant opportunistic organisms that cause nosocomial infections (1). All *Enterobacter* spp. strains harbor inducible chromosomally encoded AmpC β-lactamase genes (serine β-lactamase, Ambler class C) and are innately resistant to penicillins and first- and second-generation cephalosporins but are susceptible to carbapenems (2, 3). AmpC expression is closely linked to the peptidoglycan recycling system and is complexly regulated by AmpR (a transcriptional regulator of the LysR family), AmpD (a cytoplasmic amidase), and AmpG (a transmembrane permease) (4, 5). The β-lactam antibiotics carbapenems, cephamycin, and clavulanic acid act as strong *ampC* inducers (6). These AmpC β-lactamase induction mechanisms are also observed in Enterobacterales such as *Klebsiella aerogenes* (formerly *Enterobacter aerogenes*) and *Citrobacter freundii* (6).

Multidrug resistance has been observed for last-resort carbapenems and has led to increased global interest in Enterobacterales in general and the carbapenem-resistant *Enterobacter cloacae* complex (ECC) in particular (7). The increasing reports of carbapenem resistance are concerning. Carbapenem resistance in *Enterobacter* spp. is conferred either through a combination of overexpression of AmpC β-lactamase and loss of outer membrane (porin) permeability, or the acquisition of the carbapenemase gene, with the latter scenario being more common (7). Carbapenemases hydrolyze most β-lactams, including carbapenems, and are classified as serine β-lactamases (Ambler class A; *Klebsiella pneumoniae* carbapenemases [KPC] type and D; OXA-48 type) or metallo-β-lactamases (MBL; Ambler class B; IMP type, VIM type, and New Delhi metallo-β-lactamase [NDM] type) (8, 9). Chromosomally encoded carbapenemase non-metallo-carbapenemase of class A (NmcA) has sporadically been reported in ECC (10, 11).

NmcA has been detected in ECCs, including *E. cloacae*, *Enterobacter asburiae*, and *Enterobacter ludwigii*, in Europe, North America, South America, and Japan (11–15). This enzyme hydrolyzes different β-lactam agents except for third-generation cephalosporin (3GC) and has particularly high hydrolytic activity against carbapenems (16, 17). NmcA is the only inducible carbapenemase, and its expression is regulated by NmcR, a transcriptional regulator similar to AmpR. *Enterobacter* spp. with NmcA also possess AmpC. We previously demonstrated that NmcA expression is regulated in a manner similar to the AmpC regulatory system and, when induced, leads to 3GC resistance (5), mainly due to *ampC* overexpression. Carbapenems (imipenem and meropenem [MEM]) and cephamycin (cefoxitin) are strong β-lactamase inducers of NmcA production, similar to AmpC production. NmcA producers are highly resistant to carbapenems because their inducers are upregulated and are potently hydrolytic to carbapenems (17).

Although NmcA genes are all encoded on chromosomes, the highly homologous carbapenemase imipenem-hydrolyzing β-lactamase (IMI) genes are encoded on plasmids or chromosomes and have been detected in *E. cloacae*, *E. asburiae*, and *Escherichia coli* isolates (10, 11, 18). The registered IMI gene variants (IMI-1–IMI-24) (19) show 82.2%–96.9% DNA sequence identity with NmcA (Fig. S1). PCR amplification is the only method described for detecting NmcA/IMI producers in clinical laboratories, which may explain why these isolates are rarely reported in clinical settings (20). Currently, the methods for detecting and identifying carbapenem-resistant strains include phenotypic methods (e.g., Carba NP test, modified Hodge test, modified carbapenem inactivation method, and lateral flow immunoassays) and genotypic methods for the precise identification of carbapenemase genes (e.g., PCR, loop-mediated isothermal amplification [LAMP] assay, and whole-genome sequencing) (21–24). Here, we developed phenotypic and genotypic methods for the identification of NmcA β-lactamases and evaluated their usefulness in clinical laboratories.

In this study, we developed a phenotyping method using a double-disk synergy test (DDST) to detect NmcA producers. Inducible AmpC producers, including *Enterobacter* spp. and *K. aerogenes*, show a “D-shaped” flattening of the inhibition zone around the 3GC disk adjacent to the cefoxitin disk in DDST (25, 26). This phenomenon results from increased AmpC β-lactamase expression induced by cefoxitin, which results in resistance to 3GC. Because wild-type *Enterobacter* spp. and *K. aerogenes* are susceptible to carbapenems, while NmcA producers are resistant to carbapenems, we used carbapenem as an inducer instead of cefoxitin to identify NmcA producers. We also evaluated the combined disc test using disks containing a combination of the β-lactamase inhibitors to identify carbapenem-resistant strains, including NmcA producers (27, 28). We used MEM disks supplemented with dissolved β-lactamase inhibitors of class A carbapenemase and AmpC β-lactamase and combined with an inhibitor of class B carbapenemase (MBL) (29). Here, we describe a multiplex DDST method that combines these phenotypic assays to detect and identify NmcA producers, other carbapenemase producers, and carbapenemase-non-producing carbapenem-resistant *Enterobacter* spp. and *K. aerogenes*.

This study also describes the development of a rapid LAMP genotyping assay to detect NmcA producers. LAMP is a simple and cost-effective assay that amplifies target sequences with high specificity and sensitivity under isothermal conditions (30). LAMP methods have been developed to detect various carbapenemase-producing bacteria, including KPC producers and NDM producers (23, 31, 32). In the present study, we designed LAMP primers to detect NmcA producers and evaluated them using control NmcA producers and high-homology carbapenemase IMI producers. Finally, we developed a multiplex DDST method and LAMP assay for the detection and identification of NmcA producers and validated their accuracy using carbapenemase-producing or carbapenem-resistant strains.

## RESULTS

### Antimicrobial susceptibility and molecular characterization of the bacterial strains

**Fig 1:**
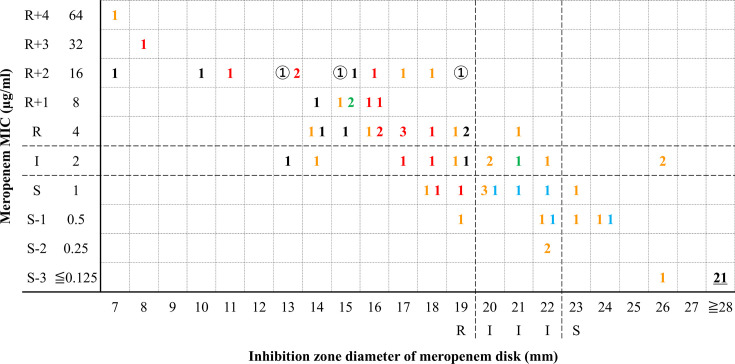
Scattergram comparing MEM MIC and disk diffusion zone diameters with 10 µg disks among *Enterobacter* spp. (*n* = 75) and *K. aerogenes* (*n* = 11) analyzed in this study**.** Dashed lines indicate the MEM MIC breakpoints (Clinical and Laboratory Standards Institute [CLSI]). Circular numbers: number of NmcA or IMI producers; pink numbers: number of IMP producers; red numbers: number of NDM producers; blue numbers: number of OXA-48-like producers; green numbers: number of KPC producers; black numbers: number of carbapenem-resistant strains due to porin loss; underlined numbers: number of MEM-sensitive isolates.

The MIC and MIC ranges of strains used in this study are shown in Table 1. NmcA and IMI producers were highly resistant to cefmetazole and MEM but susceptible to piperacillin and 3GC (cefpodoxime and cefotaxime). Almost all the KPC (3/3) and NDM producers (14/16) were resistant to MEM. Some IMP producers (15/27) and no OXA-48 producers (0/5) were susceptible to MEM. All of the carbapenemase-non-producing carbapenem-resistant strains, due to AmpC β-lactamase production and porin loss, were resistant to MEM. The results of the comparisons of MEM MIC values and disk diffusion zone diameters with a 10 µg disk were shown in Fig. 1. All carbapenemase-producing and carbapenem-resistant strains (*n* = 65) were positive for the screening cut-off value (MEM < 28 mm with disk diffusion or MIC >0.125 µg/mL) of the multiplex DDST algorithm, while other carbapenem-sensitive strains (*n* = 21) were all negative. NmcA producers were resistant to MEM, with MICs of 16 µg/mL, and were also resistant according to the disk diffusion test, with inhibitory zones of 19 mm. All other MEM-resistant (MIC ≥4 µg/mL) strains also showed resistance in the disk diffusion tests, with inhibitory zones ≤19 mm. The MEM-susceptible (MIC ≤1 µg/mL) strains included NDM (*n* = 2), IMP (*n* = 12), and OXA-48 (*n* = 5) type producers; the interpretation of the results of these tests was susceptible (24 mm), intermediate (20–22 mm), and resistant (18–19 mm).

**TABLE 1:** Detection of NmcA producers using multiplex DDST and LAMP assays for *Enterobacter* spp. and *K. aerogenes*, and their antimicrobial susceptibilities

Species^*a*^	Resistant mechanisms (Ambler class)	Strain or no. of strains	No. of correctly identified by multiplex DDST^*c*^	Inhibition zone diameter (mm) or effect with^*c*^					Detection of *bla*_NmcA_ by LAMP^*f*^	MICs or MIC range (µg/mL)^*c*^				
				MEM	MEM/APB	MEM/CLX	SMA^*e*^	CPD^*e*^		PIP	CPD	CTX	CMZ	MEM
*E. ludwigii*	NmcA (A)	NR1491	1	19	26^*d*^	20	No	D-shaped	＋	4	4	0.25	>256	16
*E. ludwigii*	NmcA (A)	NR3901	0	13	21^*d*^	15	No	No	＋	128	>256	32	>256	16
*Enterobacter roggenkampii*	IMI-16 (A)	NR5612	1	15	23^*d*^	18	No	D-shaped	＋	2	1	0.125	256	16
*E. coli*	IMI-2 (A)	NR4460	NA	20	27	24	No	No	＋	8	8	0.5	2	16
*E. coli*	IMI-18 (A)	NR5611	NA	25	32	25	No	No	＋	4	1	≦0.063	2	8
ECC	KPC-2 (A)	3	3	15–21	21–26^*d*^	15–22	No	No	–	256–>256	64–>256	16-256	2–>256	2–8
ECC	NDM-1 (B)	11	11	11–19	11–20	13–21	Expanded	No	–	64–>256	>256	256–>256	128–>256	1–16
*E. asburiae*	NDM-1 (B)	1	1	8	10	9	Expanded	No	–	>256	>256	>256	>256	32
ECC	NDM-7 (B)	4	4	16–18	16	16–19	Expanded	No	–	128–256	>256	128–>256	>256	4
ECC	IMP-1 (B)	16	16	7–26	7–27	10–27	Expanded	No	–	2–>256	32–>256	8–>256	128–>256	≤0.06–64
*K. aerogenes*	IMP-1 (B)	1	1	22	23	23	Expanded	No	–	8	64	16	>256	0.5
ECC	IMP-6 (B)	10	10	14–26	13–26	15–26	Expanded	No	–	256–>256	256–>256	64–>256	256–>256	0.25–16
ECC	OXA-48 (D)	4	4	21–24	21–24	21–24	No	No	–	256–>256	1–128	1–32	4–>256	0.5–1
*Enterobacter hormaechei*	OXA-232 (D)	1	1	20	20	22	No	No	–	>256	256	64	>256	1
ECC	Porin loss	5	5	7–19	18–24^*d*^	20–26^*d*^	No	No	–	8–64	256–>256	32–256	>256	2–16
*K. aerogenes*	Porin loss	6	6	13–19	20–25^*d*^	20–26^*d*^	No	No	–	32–256	>256	128–>256	>256	2–16
ECC	CTX-Ms^*b*^ (A)	5	5	31–35	30–35	30–35	No	No	–	128–>256	>256	64–256	64–>256	≤0.06
ECC	–	9	9	28–36	30–35	29–34	No	No	–	1–4	1–16	0.125–1	2–>256	≤0.06–0.125
ECC	–	3	3	28–30	29–30	27–30	No	D-shaped	–	0.5–2	1–8	0.125–1	128–>256	–0.06–0.125
*K. aerogenes*	–	4	4	28–31	30–31	30–31	No	No	–	4–64	0.5–256	0.125–16	64–>256	≤0.06

^*a*^ ECC means *E. cloacae* complex with the six species including *E. cloacae*, *E. asburiae*, *Enterobacter hormaechei*, *Enterobacter kobei*, and *E. ludwigii*.

^*b*^ CTX-Ms include CTX-M-2 (1) and CTX-M-27 (4).

^*c*^ Abbreviation: NA, not adapted; APB, 3-aminophenylboronic acid; CLX, cloxacillin; SMA, sodium mercaptoacetic acid; CPD, cefpodoxime; PIP, piperacillin; CTX, cefotaxime; CMZ, cefmetazole.

^*d*^ Inhibition zone of the strains was expanded by more than 5 mm compared to MEM.

^*e*^ No, no synergy was observed near the SMA or CPD disk; expanded, inhibition zone was expanded near the SMA disk; D-shaped, inhibition zone was D-shaped near the MEM disk.

^*f*^ ＋, all isolates were positive for LAMP assay; –, all isolates were negative for LAMP assay.

### Performances of the combination disc tests for identifying NmcA producers

**Fig 2:**
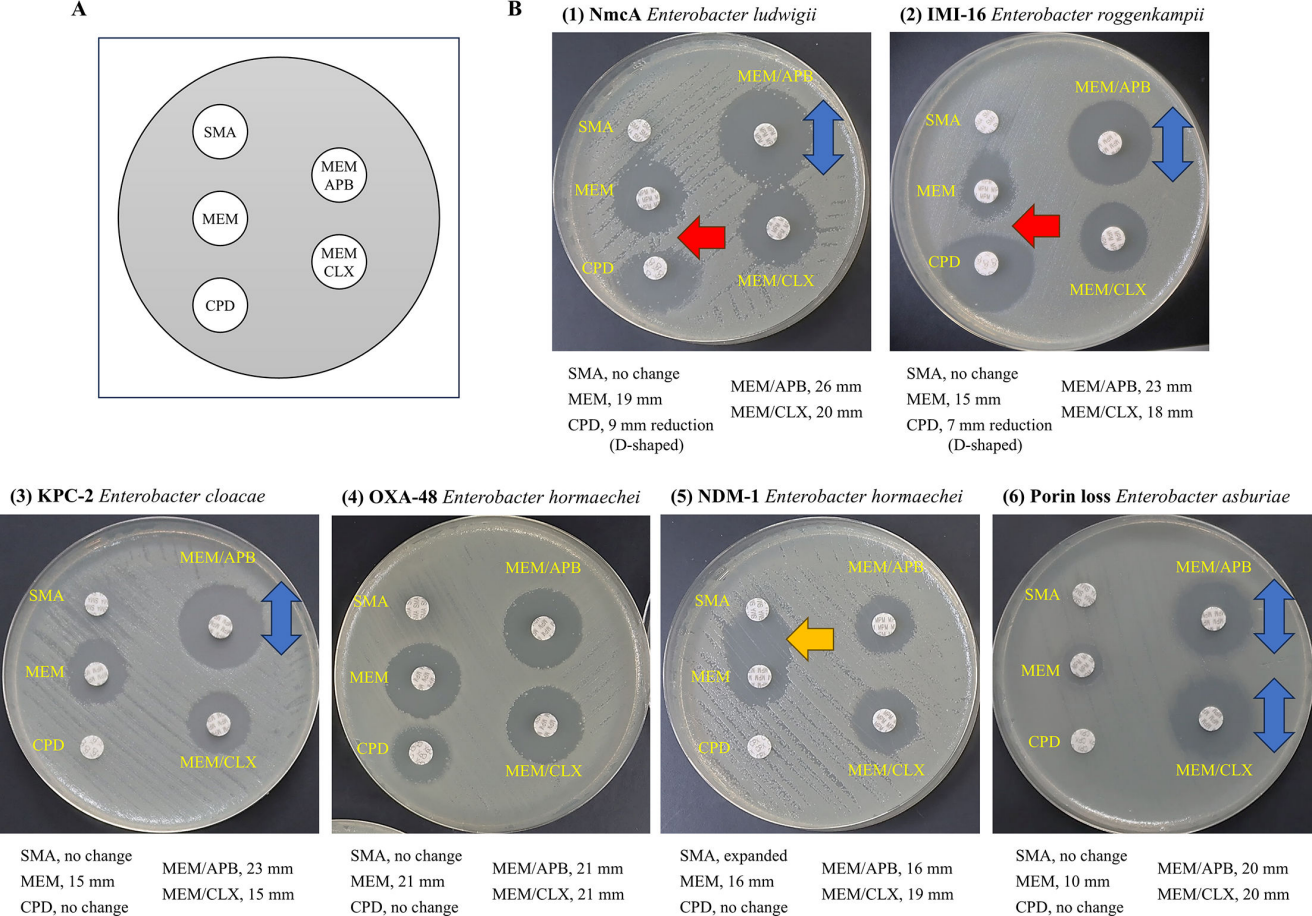
DDST for identifying the carbapenem resistance mechanisms of *Enterobacter* spp. (**A**) Disk placement of the multiple DDST. MEM (10 µg), APB (300 µg), CLX (750 µg), SMA (3 mg), and CPD (10 µg) were used. SMA and CPD were placed 20 mm (center to center) from MEM. (**B**) An example of a DDST positive result. (1 and 2) NmcA-harboring *E. ludwigii* and IMI-16-harboring *Enterobacter roggenkampii* show a distinct zone of growth inhibition that appears in MEM containing APB, while a D-shaped inhibition zone of CPD by MEM. (3) KPC-2-harboring *E. cloacae* show a distinct zone of growth inhibition in MEM containing APB. (4) OXA-48-harboring *Enterobacter hormaechei* shows no change in the inhibition zone of MEM containing APB or CLX. (5) NDM-1-harboring *Enterobacter hormaechei* shows no change in the inhibition zone of containing APB or CLX, while a distinct growth inhibition zone appears between the MEM and SMA. (6) *E. asburiae* with porin loss shows a distinct zone of growth inhibition in MEM containing APB or CLX. Red arrow: regions with a D-shaped inhibition zone of CPD by nearby MEM; blue arrows: expanded inhibition zone of MEM containing APB or CLX; yellow arrow: enhanced synergistic inhibition zone of MEM by the presence of SMA. APB (3-aminophenylboronic acid); CLX (cloxacillin); CPD (cefpodoxime); SMA (sodium mercaptoacetic acid). The inhibition zone diameters for MEM with or without inhibitors and the effects of SMA or CPD are shown below the figure.

DDST was performed by placing a MEM disk 20 mm (center to center) from a cefpodoxime disk to detect NmcA producers. The DDST results were presented in Fig. 2; Fig. S2. NmcA-producing NR1491 was non-resistant to cefpodoxime (MIC: 4 µg/mL) and showed a large inhibition zone (27 mm). Furthermore, D-shaped inhibition zones around the cefpodoxime, where the inhibition zone adjacent to the MEM disk was blunted, were observed only for NmcA/IMI producers. This D-shaped inhibition zone was also observed when a cephamycin disk (30 µg cefoxitin or 30 µg cefmetazole) was used instead of carbapenem (Fig. S2). This D-shaped inhibition zone was not observed in other carbapenemase producers.

### Accuracy of disc diffusion method for the identification of NmcA producers

We evaluated the accuracy of the multiplex DDST using five antibiotic disks for identifying NmcA and other carbapenemase producers (Table 1). NmcA and IMI producers showed a D-shaped inhibition zone for cefpodoxime discs and an expanded inhibition zone for MEM discs by APB, which we interpreted as expected NmcA/IMI producers, except for NR3901, an AmpD mutant strain. NR3901 was highly resistant to most β-lactams, including 3GC, and had a narrow inhibition zone for cefpodoxime, resulting in a lack of a D-shaped inhibition zone. Regarding other carbapenemase producers, all KPC producers showed an expanded inhibition zone of MEM discs by APB, as expected. The OXA-48 type producers showed no differences in the inhibition zone of the MEM disk containing β-lactamase inhibitors near the SMA disk. Similarly, the inhibition zones for IMP and NDM showed no differences for the MEM disk containing β-lactamase inhibitors, while the zones were expanded near the SMA disk. All of the carbapenemase-non-producing carbapenem-resistant strains, including *Enterobacter* spp. and *K. aerogenes*, show expanded inhibition zones for the MEM disk containing β-lactamase inhibitors (APB and CLX). These expanded inhibition zones resulted from AmpC β-lactamase inhibition. Among carbapenem-sensitive strains (*n* = 21), three showed a partial D-shaped inhibition zone for the cefpodoxime discs, but all the strains were negative for the screening cut-off (MEM < 28 mm with disk diffusion or MIC > 0.125 µg/mL) of the multiplex DDST algorithm. The multiplex DDST developed in the present study showed a 98.8% (85/86) accuracy.

### Sensitivity and specificity of the LAMP method for detecting NmcA producers

**Fig 3:**
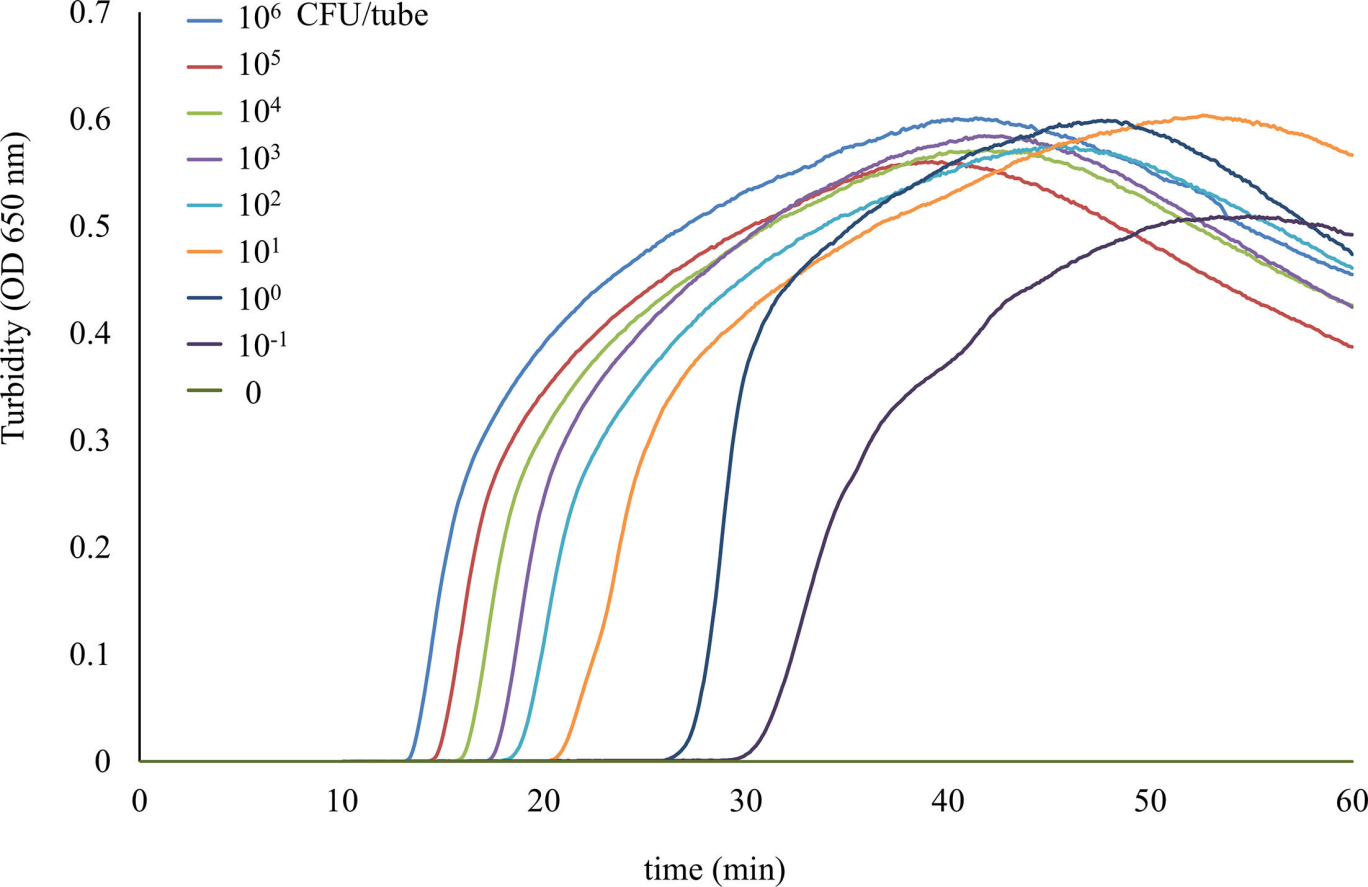
Sensitivity of the LAMP reaction for the detection of *bla*_NmcA_. DNA was extracted from serially diluted *E. ludwigii* NR1491 cells and used as templates for the reactions. The LAMP reactions were monitored in real-time using a turbidity assay and a Loopamp Real-time Turbidimeter. Shown from left to right are the curves of decreasing bacterial concentration, from 10^6^ to 10^−1^ CFU per tube.

The optimal temperature for efficient LAMP-based amplification for detecting *bla*_NmcA_ was 63°C (Fig. S4). Regarding the detection limit of the LAMP assay, the comparison of the results for serial 10-fold dilutions of DNA extracted from cultivated *E. ludwigii* (*E. cloacae* complex) NR1491 cells with PCR results (Fig. 3) showed that the LAMP assay amplified the 10^6^ CFU/tube in 16 min. Moreover, the detection limit was 10^−1^ CFU per tube in 33 min, which was 100-fold more sensitive than the PCR assay for *bla*_NmcA_ detection.

We evaluated the sensitivity and specificity of the LAMP assay for the detection of NmcA/IMI producers (*n* = 5) by assessing their reactivity with strains producing or not producing other types of carbapenemases (*n* = 83) (Table 1). The LAMP assay specifically detected NmcA and IMI producers (IMI-2, IMI-16, and IMI-18) that are highly homologous to NmcA. The numbers of bases mismatched with the primer sequences are as follows: IMI-2, F3 (1/22), FIP (1/44), BIP (1/40), B3 (1/23), LF (0/22), and LB (0/21); IMI-16, F3 (0/22), FIP (1/44), BIP (0/40), B3 (2/23), LF (0/22), and LB (1/21); and IMI-18, F3 (0/22), FIP (1/44), BIP (0/40), B3 (3/23), LF (0/22), and LB (1/21; Fig. S3). Furthermore, we observed no cross-reactivity with other types of carbapenemase producers, carbapenemase non-producers, or negative controls (distilled water). Thus, the sensitivity and specificity of the LAMP assay were 100% (5/5) and 100% (83/83), respectively.

## DISCUSSION

In this study, we developed DDST and a LAMP assay to detect NmcA/IMI producers using carbapenem-resistant strains and carbapenemase producers. NmcA and IMI, which are highly homologous to NmcA, are inducible carbapenemases. Strains producing these compounds are resistant to β-lactams, including carbapenem; however, these isolates are susceptible to 3GC (16, 33). All the tested NmcA/IMI-producing strains in the present study were highly resistant to MEM but susceptible to 3GC, except for NR3901, an AmpD mutant strain that is resistant to most β-lactams. We designed the DDST in the present study to evaluate the induction mechanism of NmcA/IMI production by gene regulation. NmcA/IMI β-lactamases, which are strong inducers, are usually produced by carbapenems and cephamycin (15). The cefpodoxime inhibition zone changed to a D-shape near MEM or cephamycin (cefoxitin and cefmetazole) disks (Fig. S2). This change in inhibition zone shape may occur due to an overproduction of AmpC β-lactamase induced by the antibiotics, resulting in cefpodoxime resistance. *Enterobacter* spp. encodes AmpC β-lactamase and the regulator gene *ampR*, whose induction may also influence production and the cefpodoxime inhibition zone. However, wild strains of *Enterobacter* spp. are generally susceptible to MEM disks, and the inhibition zone is too wide to identify a distinct D-shaped inhibition zone for cefpodoxime. Even if a D-shaped inhibition zone is identified, it does not meet the criteria for NmcA/IMI because it is sensitive to MEM. Furthermore, we also evaluated IMI-producing *E. coli* that did not encode the inducible AmpC β-lactamase and *ampR*. These strains did not show D-shaped inhibition zones for cefpodoxime disks, similar to NmcA/IMI-producing *Enterobacter* spp. These results suggest that the D-shaped inhibition zone results from AmpC β-lactamase induction. No D-shaped inhibition zone was observed in other carbapenemase producers that were not resistant to cefpodoxime. These results suggest that NmcA β-lactamase induction may also affect the D-shaped inhibition zone because NmcA is co-regulated in a similar manner to the AmpC regulatory system (5). Although a cefpodoxime disc was used in this study, the same phenomenon was observed when cefotaxime was used (data not shown).

We also evaluated the accuracy of the multiplex DDST for identifying NmcA producers, other carbapenem-resistant strains, and carbapenemase producers. All class A carbapenemase (NmcA/IMI and KPC) producers were inhibited by only APB. KPC producers showed a narrow cefpodoxime inhibition zone; however, no D-shaped inhibition zone was observed. NmcA/IMI producers showed a D-shaped inhibition zone for cefpodoxime, except for NR3901, an AmpD mutant strain. NR3901 resulted in a constant overproduction of NmcA and AmpC due to the AmpD mutation and was highly resistant to most β-lactams (15). Although AmpD-mutant NmcA producers have not been reported in clinical isolates, it is difficult to identify them as NmcA producers using this detection method. In addition, AmpR-mutant strains have a higher MIC for cefpodoxime and a narrower inhibition zone (15); therefore, detection by this method, as well as the AmpD mutant strain, is challenging. Since NmcA producers, whether wild-type or mutant, tend to be highly resistant to carbapenems, screening combining this MIC feature and this method may be effective. No class B carbapenemase (NDM and IMP) producers were inhibited by any β-lactamase inhibitors, which showed expanded zones near the SMA disk, as expected. VIM-producing *Enterobacter* spp. were not used in this study; however, the results of the VIM-producing *C. freundii* strain were correctly applied to the criteria for class B carbapenemase (data not shown). This method is likely to also effectively identify VIM producers as class B carbapenemase producers. OXA-48 type producers showed an intermediate interpretation of MEM disks and were not inhibited by any β-lactamase inhibitors. All wild strains of *Enterobacter* spp. showed a sensitive interpretation of the MEM disks and were not inhibited by any β-lactamase inhibitors. Identification of OXA-48 type producers can be difficult and may require the use of temocillin, to which they are resistant, for a more accurate identification (28). The developed method identified carbapenemase-non-producing carbapenem-resistant strains due to porin loss, which were inhibited by both β-lactamase inhibitors (APB and CLX). AmpC β-lactamase was inhibited by APB and CLX, and the inhibition zone of MEM was expanded in combination with the inhibitors. As this method was applied to *K. aerogenes*, it is also likely applicable to other AmpC-producing Enterobacterales. The carbapenem resistance mechanisms in carbapenem-resistant *K. aerogenes* are mainly linked to the production of chromosomal AmpC β-lactamases along with changes in membrane permeability through porin regulation (34–36). Thus, the methods described in this study are also expected to be effective against other bacterial species. The accuracy of the DDST was high; therefore, it may be effective for use in clinical laboratories. We also evaluated the use of ertapenem instead of MEM disks (data not shown). The accuracy of the multiplex DDST using ertapenem disks was 87.2% (75/86); thus, a MEM disk may be suitable for this method. To our knowledge, no previous studies have reported the use of DDST specifically designed to detect NmcA or IMI producers. Although DDST has been widely applied to detect extended-spectrum β-lactamases (ESBLs) and MBLs, its application to inducible class A carbapenemases such as NmcA and IMI is novel. Therefore, the present study represents a first attempt to evaluate DDST as a phenotypic tool for detecting these rare but clinically relevant carbapenemases.

We also developed a LAMP-based amplification to detect *bla*_NmcA_. This method detected NmcA producers rapidly (within 16 min) and was 100-fold more sensitive than PCR. Although this method was designed to detect NmcA producers, it can also detect IMI producers (IMI-2, IMI-16, and IMI-18) that are homologous to NmcA. Although the primers did not exactly match the corresponding regions, this method was able to amplify these genes (Figs. S1 and S3). The numbers of mismatched bases between the IMI genes and primer sequences were as follows: F3 (1–6/22), FIP (1–3/44), BIP (0–8/40), B3 (0–5/23), LF (0–2/22), and LB (0–1/21). Because most of the genes showed high homology to the primers, they were expected to detect other types of IMI. However, IMI-5 showed the lowest homology (82.23%) to NmcA and also had a mismatch at the 3' end of the F3 primer; thus, it was not amplified. This method demonstrated excellent rapidity, high sensitivity, and high specificity for detecting NmcA genes and is expected to be utilized in clinical microbiology laboratories.

Among phenotypic methods for detecting carbapenemase-producing bacteria, NmcA producers were all positive using the modified carbapenem inactivation method, Carba NP test, and modified Hodge test (Data not shown). A lateral flow immunoassay (NG-Test CARBA 5) showed negative results, indicating that it is not one of the five most common carbapenemase producers (KPC, IMP, NDM, VIM, and OXA-48) (22). Neither result can show that the bacteria are NmcA producers, and the carbapenemase gene cannot be accurately typed. Therefore, we propose a screening method combining these detection methods. For example, positive mCIM and negative NG-Test CARBA 5 findings indicate that the bacteria produce uncommon carbapenemases such as NmcA (22). The DDST and LAMP methods could be used to more appropriately detect NmcA producers against such resistant strains.

The DDST developed in this study offers a simple and cost-effective method for the phenotypic screening of NmcA producers. Given that many clinical laboratories lack advanced molecular or mass spectrometry tools, the DDST provides a practical alternative for the initial identification of rare inducible carbapenemases such as NmcA or IMI, especially in low-resource settings. Furthermore, its reliance on observable zone changes makes it easily interpretable and potentially applicable to routine susceptibility testing. The LAMP assay offers rapid (<16 min), highly sensitive, and specific detection of *bla*_NmcA_, with the additional ability to detect some IMI variants. This speed is critical in clinical settings where prompt identification of carbapenemase producers can inform treatment decisions and guide infection control measures (23, 37). The LAMP assay may also be adapted for point-of-care testing and outbreak investigations in hospital environments, offering a valuable addition to diagnostic workflows. As the prevalence of NmcA remains low, early detection and establishment of reliable diagnostic methods are important for monitoring potential future spread. These findings may help integrate DDST and LAMP into clinical microbiology laboratories as complementary tools for carbapenemase surveillance.

This study has some limitations. First, since the effectiveness of multiplex DDST was evaluated on a limited number of strains, generalizations should be made with caution. The current methods for detecting NmcA producers are insufficient, and the few relevant reports have also described the challenges of obtaining a sufficient number of samples. The strain used in this study is similar to the reported reference strain and is expected to yield similar results to other NmcA producers; however, it may not discriminate well from AmpD mutant strains. Therefore, screening focusing on highly carbapenem-resistant strains, a characteristic of NmcA producers, may be more effective. Furthermore, the developed LAMP method can be used as a complementary technique. Therefore, future large-scale studies using a larger number of strains are required. Second, we developed a multiplex DDST assay to detect carbapenemase in *Enterobacter* spp. Although inducible AmpC production is an important factor in this method, it is difficult to detect and identify IMI producers in *E. coli* that do not produce inducible AmpC. Furthermore, it is difficult to detect AmpD-mutant NmcA producers because they are converted to overproduction rather than induction. Third, the LAMP assay successfully detected NmcA and some types of IMI producers; however, some types of IMI producers (e.g., IMI-5) were not detected. Therefore, the applicability of this method to various IMI-producing strains requires further evaluation. Finally, the behavior of carbapenem-resistant strains harboring multiple carbapenemases has not been evaluated and is a subject for future studies.

In conclusion, we developed and described DDST and LAMP assays that detected and identified NmcA producers, respectively, with high accuracy, sensitivity, and specificity. The DDST assay effectively identified NmcA-producing *Enterobacter* spp., which showed “D-shaped” inhibition zones using 3GC and carbapenem disks. This method is expected to be effective for induced AmpC producers. The LAMP assay rapidly detected NmcA as well as IMI producers.

## MATERIALS AND METHODS

### Bacterial strains

NmcA-producing *E. ludwigii* NR1491 and NR3901 were used as reference strains (15). To evaluate the accuracy of the developed DDST and LAMP assays for detecting and identifying NmcA producers, we also used well-characterized *Enterobacter* spp. and *K. aerogenes* strains stocked in our laboratory (86 strains) isolated from hospital patients and the environment (river water or hospital sewage), including some of the strains used in previous studies (22) (Table 1). The species were identified using matrix-assisted laser desorption ionization time-of-flight mass spectrometry (VITEK MS, Sysmex bioMérieux Co., Ltd., Tokyo, Japan). Carbapenemase-producing strains harboring the KPC (*n* = 3), NDM (*n* = 16), IMP (*n* = 27), and OXA-48-like (*n* = 5) genes were used (22, 38). Additionally, CTX-M-producing (*n* = 5) and carbapenem-susceptible strains (*n* = 16) were also used. For carbapenemase non-producing strains resistant to carbapenem due to porin deficiency, we used *Enterobacter* spp. (*n* = 5) and *K. aerogenes* (*n* = 6). Carbapenemase IMI, which has very high homology to NmcA-producing bacteria, IMI-2-producing *E. coli* NR4460, IMI-18-producing *E. coli* NR5611, and IMI-16-producing *Enterobacter roggenkampii* NR5612, was used to evaluate the sensitivity and specificity of the developed LAMP assay.

### Antimicrobial susceptibility testing and identification of resistance genes

We evaluated the MIC of the strains using the agar dilution method according to the Clinical and Laboratory Standards Institute guidelines M100 35th Edition (39). *E. coli* ATCC 29522 was used for quality control. The resistance genes of the strains (carbapenemase, extended-spectrum β-lactamase, and plasmid-mediated AmpC β-lactamase) were determined using PCR and DNA sequencing (40–42). We detected carbapenemase non-producing carbapenem-resistant strains according to the presence of alterations or losses of outer membrane porins (OmpC and OmpF) by DNA sequencing and the absence or reduced expression of the porin gene by quantitative reverse transcription-PCR (43).

### Disc diffusion method and interpretation

To identify NmcA producers, we performed confirmatory induction tests using the disk diffusion method (25, 26). The screening cutoff value of MEM MIC > 0.125 µg/mL was determined based on previous studies that have reported low-level carbapenem resistance in Enterobacterales (28). MEM disks (Eiken Chemical, Japan) were used as inducers instead of cefoxitin and were placed adjacent to the cefpodoxime disks (Eiken Chemical). Inducible NmcA producers show a “D-shaped” flattening of the inhibition zone around the 3GC disk adjacent to the MEM disk.

To identify carbapenem resistance mechanisms, we performed multiplex DDST using MEM disk, cefpodoxime disk, and 3 mg sodium mercaptoacetic acid disk (SMA, Eiken Chemical) as MBL inhibitor, MEM disks supplemented with dissolved β-lactamase inhibitors: 300 µg of 3-aminophenylboronic acid (APB, Sigma, USA) as an inhibitor of class A carbapenemase and AmpC β-lactamase, and 750 µg of cloxacillin (CLX, Sigma) as an inhibitor of AmpC β-lactamase (27–29, 39). APB was dissolved in dimethyl sulfoxide (FUJIFILM Wako Pure Chemical Corporation, Osaka, Japan), while CLX was dissolved in sterile water. The disks were dried at room temperature for 30 min before use. The strains were adjusted to a 0.5 McFarland inoculum and spread on Mueller-Hinton agar plates (Eiken Chemical). The DDST was performed by placing the SMA and cefpodoxime disks in a straight line 20 mm (center-to-center) from the MEM disk (Fig. 2A, Fig. S2). The distance of 20 mm (center-to-center) was chosen based on previous studies using DDST for carbapenemases and ESBL detection (44–46). Additionally, preliminary experiments with distances of 15, 20, and 25 mm demonstrated that 20 mm provided the most distinct D-shaped inhibition zone, thus optimizing the sensitivity and specificity of the test. The MEM disks in combination with the inhibitors APB and CLX were also placed on the plate (Fig. 2A).

**Fig 4:**
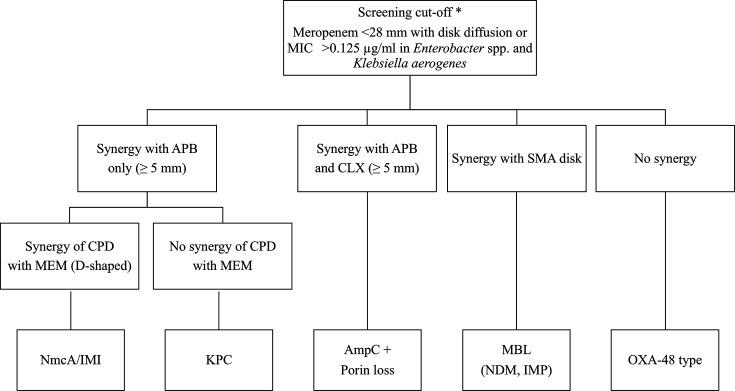
Algorithm interpretation of results obtained with the multiplex DDST for identifying the carbapenem resistance mechanisms in *Enterobacter* spp. and *K. aerogenes***.** The algorithm for the identification of CRE/CPE in *Enterobacter* spp. and *K. aerogenes* has been modified from that proposed by Miriagou et al. (27). CRE, carbapenem-resistant Enterobacterales; CPE, carbapenemase-producing Enterobacterales; APB, 3-aminophenylboronic acid (inhibitor of class A carbapenemase and AmpC); CLX, cloxacillin (inhibitor of AmpC); SMA, sodium mercaptoacetic acid (inhibitor of MBL).

The carbapenem resistance mechanisms were interpreted according to the decision algorithms presented in Fig. 2B and Fig. 4. A “D-shape” positive was defined as a decrease of ≥3 mm in the zone diameter around the cefpodoxime disk adjacent to the MEM disk. An increase of ≥5 mm in the zone diameter around disks containing β-lactamase inhibitors, compared to the disk with MEM alone, was considered a positive result for APB and CLX. Positivity for APB alone indicated the production of class A carbapenemase. NmcA/IMI producers, class A carbapenemase, were considered APB positive, with a D-shaped inhibition zone for cefpodoxime by MEM. Positivity for both APB and CLX indicated the production of AmpC β-lactamase. MEM, APB-, and CLX-positive strains were carbapenemase-non-producing, carbapenem-resistant strains with porin loss. MBL producers (NDM and IMP) showed no changes when MEM was combined with APB and CLX, whereas a distinct growth inhibition zone appeared between MEM and SMA. The OXA-48-type producers, class D carbapenemases, showed no change when MEM was combined with APB and CLX, as well as around the SMA and cefpodoxime disks.

### LAMP primer design and assay for detecting NmcA producers

We designed LAMP primers to recognize *bla*_NmcA_ using PrimerExploreV4 (https://primerexplorer.eiken.co.jp/e/index.html) as described previously (23). The set of outer primers (F3 and B3), inner primers (FIP and BIP), and loop primers (LF and LB, to accelerate the reaction) were designed specifically to recognize *bla*_NmcA_ (Fig. S3). The primer sequences were as follows: F3, 5′-CTGGGTAACATACTTAGTGAAC-3′; B3, 5′-CGTTTTTTGTTGTGTATACAGAA-3′; FIP, 5′-GTACGCTAGCACGAATACGCGATGAAAAGGAAACCTATCAGACA-3′; BIP, 5′-GCGATAAAACTGGTAGTTGCGGATAAGAGGAGCCCGGTTC-3′; LF, 5′-CGGTTGTGTTACCCTTTAACCA-3′; and LB, 5′-TACGGCAAATGATTATGCGGT-3′. The *bla*_NmcA_ reference sequence was obtained from the DNA sequence of *E. ludwigii* isolate NR1491 (GenBank accession number LC482123) (15). The LAMP reactions were performed using the Loopamp DNA Amplification Kit (Eiken Chemical) according to the manufacturer’s instructions. The reactions were carried out at the optimal temperature for 60 min; the optical density was recorded at 650 nm every 6 s using a Loopamp Real-time Turbidimeter (LoopampEXIA; Eiken Chemical). To determine the optimal temperature, the LAMP reaction was initially performed at a range of temperatures from 58°C to 68°C. The NmcA-producing *E. ludwigii* isolate, NR1491, was used as a positive control. The boiling method was used to extract total bacterial DNA, in which the cells were heated at 99°C for 10 min and centrifuged at 10,000 rpm for 5 min to collect the supernatants.

### Accuracy, sensitivity, and specificity of the multiplex DDST and LAMP assay

We evaluated the accuracy of the multiplex DDST using 86 strains of carbapenemase-producing or non-producing *Enterobacter* spp. and *K. aerogenes* (Table 1). The performance of the disc method in detecting NmcA producers was evaluated using a genotypically defined carbapenem resistance mechanism as a reference standard. We also used *K. aerogenes* as a control carbapenemase non-producing carbapenem-resistant strain due to porin deficiency. We calculated the accuracy according to the number of correctly identified bacteria in the resistance mechanism. For the LAMP assay, serial dilutions (10^−1^–10^6^ CFU per tube) of cultured *E. ludwigii* NR1491 were used to determine the detection limit of *bla*_NmcA_ detection. PCR amplification targeting *bla*_NmcA_ was also performed using serial dilutions, as described previously, to allow a sensitivity comparison (20). We evaluated the detection sensitivity and specificity of the LAMP assay using the reference strain *E. ludwigii* isolate NR1491, 84 strains of carbapenemase-producing or non-producing *Enterobacter* spp. and *K. aerogenes*, and three IMI-producing strains (IMI-18-producing ECC, and IMI-2 or IMI-16-producing *E. coli*). We calculated sensitivity and specificity according to the numbers of true-positive and true-negative bacteria, respectively.

## Data Availability

Data will be made available on request.

## SUPPLEMENTAL MATERIAL

Supplemental material spectrum.02675-24-s0001.pdf Fig. S1 to S4.

ASM does not own the copyrights to Supplemental Material that may be linked to, or accessed through, an article. The authors have granted ASM a non-exclusive, world-wide license to publish the Supplemental Material files. Please contact the corresponding author directly for reuse.
