# Supplementary material for: Development of phenotypic and genotypic methods for the identification of NmcA β-lactamases in carbapenem-resistant Enterobacter spp. and Klebsiella aerogenes
Source: Microbiol Spectr. 2025 Jul 31;13(9):e02675-24. doi: 10.1128/spectrum.02675-24 (PMC12403657; doi:10.1128/spectrum.02675-24)
Supplement: Supplemental material — Fig. S1 to S4. [file spectrum.02675-24-s0001.pdf]

# Supplementary materials

## Development of phenotypic and genotypic method for identification of NmcA β-lactamases in carbapenem resistant *Enterobacter* spp. and *Klebsiella aerogenes*

Ryuichi Nakano,[a,*] Akiyo Nakano,[a] Yuki Suzuki,[a] Kazuya Narita,[b] Takahiro Sekine,[a] Hisakazu Yano[a]

[a] Department of Microbiology and Infectious Diseases, Nara Medical University, 840 Shijo-cho, Kashihara, Nara, 6348521, Japan

[b] Division of Central Clinical Laboratory, Iwate Medical University Hospital, 1-1-1 Idaidori, Yahaba-cho, Shiwa-gun, Iwate, 0283694, Japan

**Contents:**

**FIG S1. Identities of NmcA and IMI β-lactamase genes.**

**FIG S2. Performances of combination disc tests for identifying NmcA producers.**

**FIG S3. Homology of nucleotide sequences of $bla_{NmcA}$ and $bla_{IMI-1}$ to $bla_{IMI-24}$ in regions used to design LAMP primers.**

**FIG S4. Optimal temperatures of the LAMP reaction for rapid amplification of $bla_{NmcA}$.**

# FIG S1

**A**

| | IMI-3 | IMI-6 | IMI-10 | IMI-17 | IMI-2 | IMI-20 | IMI-16 | IMI-9 | IMI-12 | IMI-18 | IMI-19 | IMI-1 | IMI-4 | IMI-14 | IMI-15 | IMI-7 | IMI-21 | IMI-13 | IMI-22 | IMI-23 | IMI-8 | IMI-24 | IMI-5 |
|---|---|---|---|---|---|---|---|---|---|---|---|---|---|---|---|---|---|---|---|---|---|---|---|
| Identity with NmcA (%) | 96.92 | 96.81 | 96.70 | 96.58 | 96.58 | 96.47 | 96.47 | 96.36 | 96.36 | 96.36 | 96.36 | 96.13 | 96.13 | 96.01 | 96.01 | 95.90 | 94.99 | 94.42 | 89.41 | 89.41 | 86.90 | 86.56 | 82.23 |

**B**

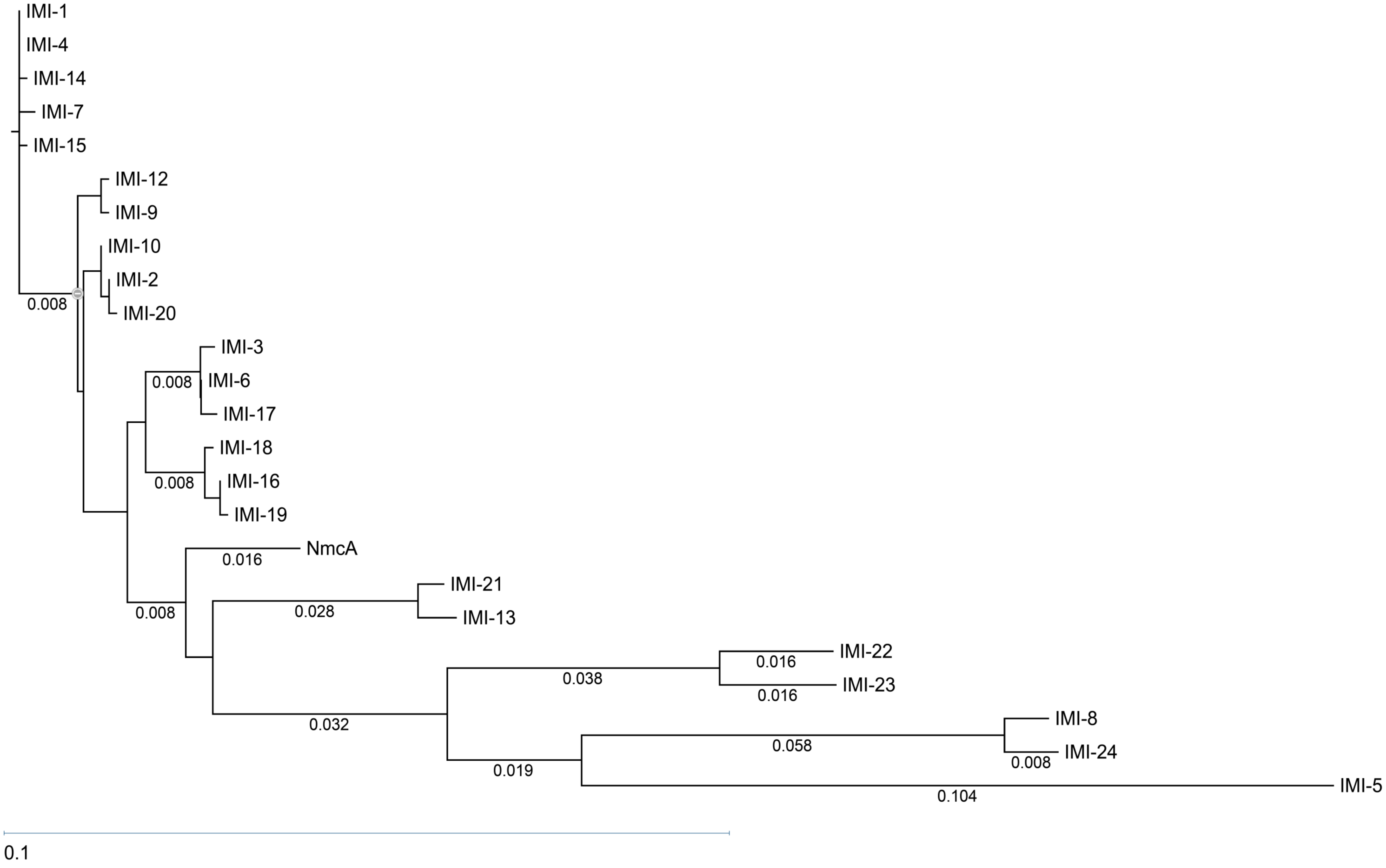


**FIG S1. Identities of NmcA and IMI β-lactamase genes.** (A) DNA sequence identities (%) of the NmcA gene with IMI genes. (B) Phylogenetic tree of NmcA and IMI genes by Clustal W using the Neighbor Joining method calculated in DNASTAR software. The bootstrap value represents the branch support. The GenBank accession number of NmcA is LC482123. The IMI DNA sequences were obtained from the GenBank accession numbers described in the β-lactamase database (www.bldb.eu [19]).

## FIG S2

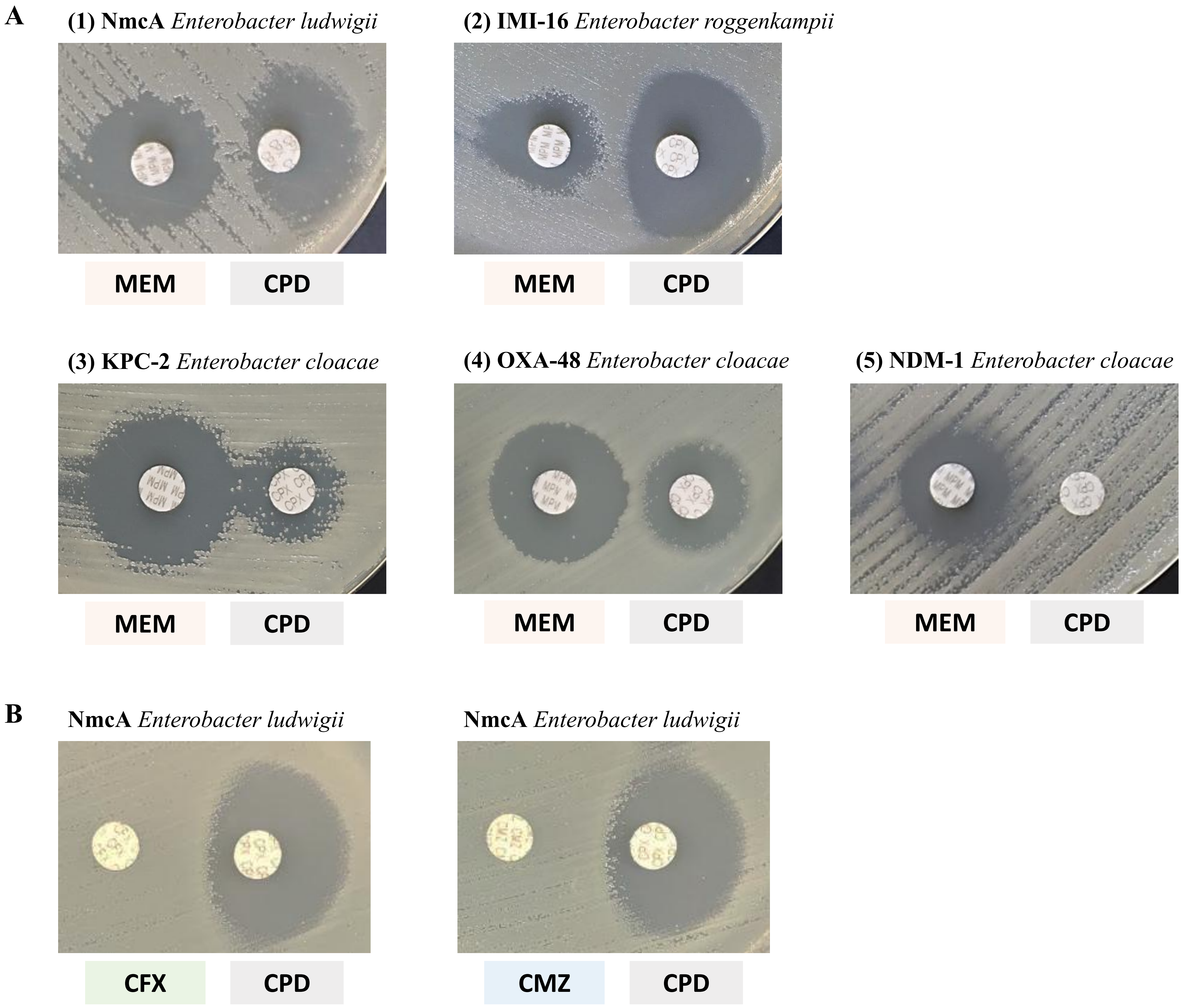


**FIG S2. Performances of combination disc tests for identifying NmcA producers.** (A) Effect of a 10 μg meropenem (MEM) disk on the inhibition zone surrounding a 10 μg cefpodoxime (CPD) disk in carbapenemase-producing isolates. A characteristic "D-shaped" zone of inhibition around the CPD disk is observed specifically in NmcA/IMI producers but not in isolates producing other types of carbapenemases. (B) A D-shaped inhibition zone was also observed using a cephamycin disk (30 μg cefoxitin (CFX) or 30 μg cefmetazole (CMZ)) instead of an MEM disk for identifying NmcA/IMI producers.

# FIG S3

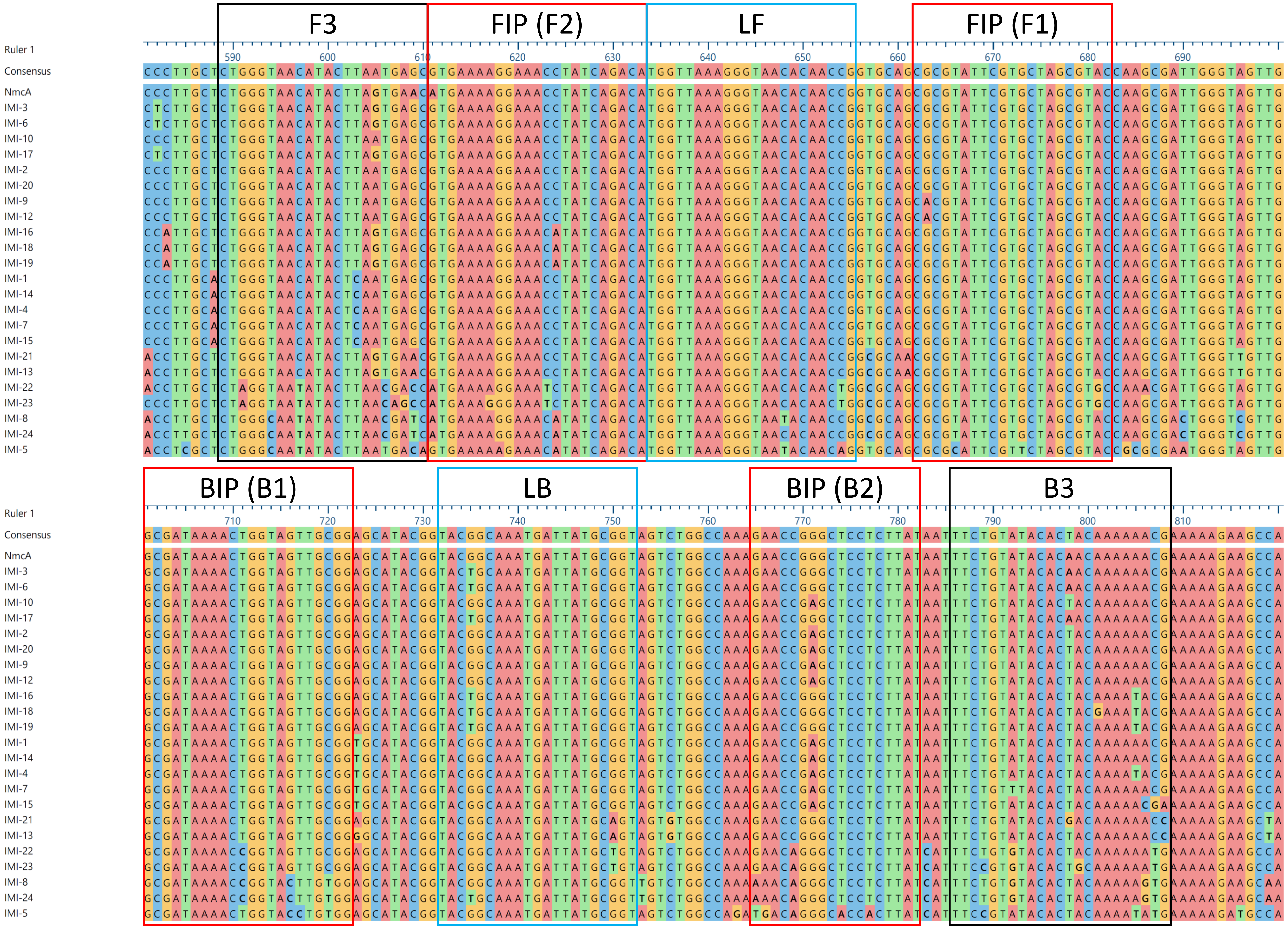


**FIG S3. Homology of nucleotide sequences of *bla*$_{\text{NmcA}}$ and *bla*$_{\text{IMI-1}}$ to *bla*$_{\text{IMI-24}}$ in regions used to design LAMP primers.** The sequences used for LAMP primers (F3, B3, FIP, BIP, and loop primers LF and LB) are indicated by squares. The inner primers (FIP and BIP) are complementary to F1 (B2) and the sense sequence of F2 (B1). Multiple sequence alignments of the *bla*$_{\text{NmcA}}$ and *bla*$_{\text{IMI}}$ were performed using DNASTAR software. NmcA GenBank accession number: LC482123. The IMI DNA sequences are obtained from GenBank accession numbers described in the β-lactamase database (www.bldb.eu [19]). The sequences are shown in the order of homology to NmcA.

**FIG S4**

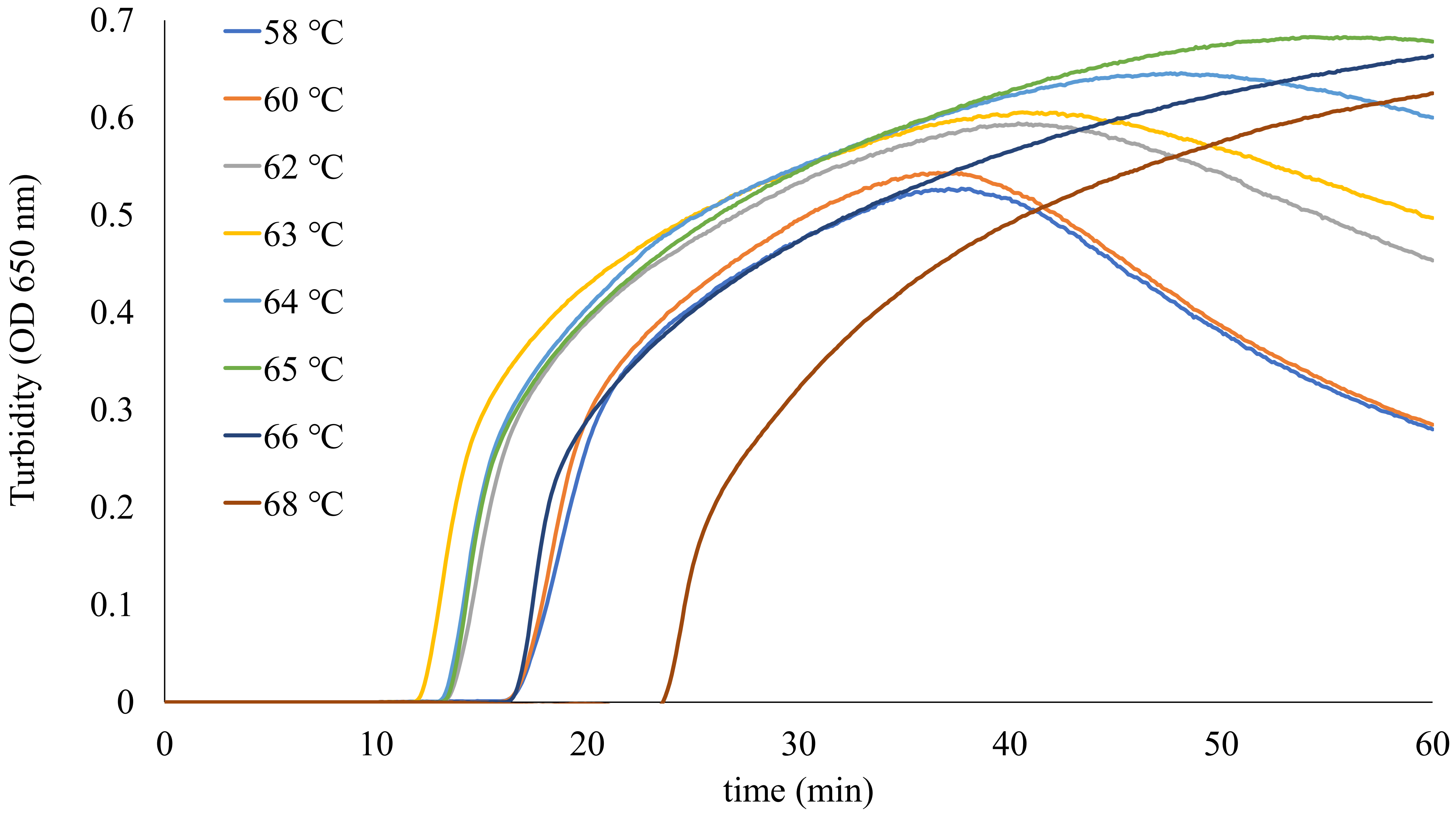


**FIG S4. Optimal temperatures of the LAMP reaction for rapid amplification of *bla*$_{\mathbf{NmcA}}$.** DNA extracted from *E. ludwigii* isolate NR1491 was used as the template in the LAMP reaction. The LAMP reaction was observed at different temperatures from 58 to 68°C.
